# Cardiovascular Risk Profile on the Island of Santiago—Cabo Verde (PrevCardio.CV Study)

**DOI:** 10.3390/life14080966

**Published:** 2024-07-31

**Authors:** Francisco Rodrigues, Kelly Mascarenhas, Júlio Rodrigues, Patrícia Coelho

**Affiliations:** 1 Sport Physical Activity and Health Research & Innovation Center (Sprint), Polytechnic Institute of Castelo Branco, 6000-084 Castelo Branco, Portugal; patriciacoelho@ipcb.pt; 2 Polytechnic Institute of Castelo Branco, 6000-084 Castelo Branco, Portugal; a2022123467@estesc.ipc.pt; 3 Instituto Nacional de Saúde Pública de Cabo Verde, Praia WF7Q+V29, Cape Verde; julio.m.rodrigues@insp.gov.cv

**Keywords:** prevalence, cardiovascular risk factors, hypertension, diabetes, pathology, Cabo Verde

## Abstract

Cerebrocardiovascular diseases are a major global public health concern, significantly impacting morbidity, mortality, and posing substantial socio-economic challenges. In Cabo Verde, non-communicable diseases have become the leading causes of morbidity and mortality. This study aimed to estimate the prevalence of risk factors for cerebrocardiovascular diseases and their association with cardiac electrical alterations in adults on Santiago Island, Cabo Verde. A cross-sectional population-based study using simple random sampling was conducted on individuals over 18 years of age. The sample size of 599 was based on Santiago Island’s 2021 population projection. Data collection occurred in October and November 2021, involving questionnaires on risk factors and cerebrocardiovascular diseases; blood pressure assessments; and capillary blood glucose measurements. The sample was predominantly female, with the 18–27 age group being the largest. Key risk factors included physical inactivity (65.1%), BMI ≥ 25 kg/m^2^ (42.6%), hypertension (32.6%), and family history of cerebrocardiovascular diseases (19.9%). Other factors were alcoholism (14.4%), hypercholesterolemia (8.3%), smoking (7.3%), diabetes (4.5%), and hypertriglyceridemia (1.3%). Notably, 9.3% had no risk factors, 27.5% had one, 36.2% had two, and 26.9% had three or more. There is a high prevalence of risk factors for cerebrocardiovascular diseases on Santiago Island, particularly among females.

## 1. Introduction

Cerebrocardiovascular diseases (CCVDs) are significant health and economic concerns, as these pathologies cause comorbidities that impact the quality of life of affected individuals and their families. They are described as disorders of the heart and blood vessels and include coronary heart disease, cerebrovascular disease, and peripheral vascular disease [1,2]. It is estimated that more than four out of five deaths from CCVD are due to heart events and strokes, with one-third of these deaths occurring in young adults [1]. According to available data, such as the global, regional, and national burden of stroke, CCVDs represent the leading cause of global mortality, with approximately 17.9 million deaths each year, accounting for 32% of all deaths worldwide, predominantly in developed and middle-income countries [3,4]. African countries, including Cabo Verde, are undergoing an epidemiological transition from communicable to non-communicable diseases, facing rapid growth in CCVDs and related risk factors [5].

According to the latest statistical reports from Cabo Verde’s Ministry of Health for the years 2017 and 2018, circulatory system diseases were the leading causes of mortality, with cerebrovascular diseases being the most prevalent in both sexes [6].

Based on the Second Non-Communicable Diseases Survey (IDNT II)—Steps Report 2020 [5], it was possible to ascertain some important aspects regarding cerebrocardiovascular (CCV) risk factors and daily habits among the adult population of Cabo Verde. It was found that 20.1% of the population has hypertension (AHT), 3.9% have diabetes, the prevalence rate of smoking is 12.5%, alcoholism 45.0%, and hypercholesterolemia 8.3%. Regarding eating habits, the population consumes an average of 3 to 4 servings of fruits/vegetables per day, on average for 4 days per week, which is below the rate recommended by the World Health Organization (WHO). The average salt consumption of Cabo Verdeans was double the recommended amount per day, and there is a high rate of consumption of processed foods. Those who declared always or frequently consuming fatty foods and/or sugary drinks accounted for 15.5% and 30.6%, respectively. CCV risk factors are described as the main determinants increasing the likelihood of developing diseases such as stroke, acute myocardial infarction (AMI), thromboses, among others. These factors can be grouped into modifiable and non-modifiable. Modifiable risk factors are those that can be controlled by adopting healthy lifestyles, medication, and management, while non-modifiable factors are related to genetics and do not directly depend on habits and lifestyles [7,8]. Among the factors, AHT plays a significant role in the development of CCVD, with an estimated 29% of the global population expected to be hypertensive by 2025 [9]. Santiago Island has the highest population density in Cabo Verde, consisting of nine municipalities with diverse characteristics, where a broad spectrum of risk factors for CCVD can be found, reflecting the country’s reality to some extent.

Santiago Island, the largest and most populous island of Cabo Verde, is home to a diverse and vibrant population. The inhabitants, known for their rich cultural heritage, are a mix of African and Portuguese descent, reflecting the island’s history of colonization and the transatlantic slave trade. The island’s capital, Praia, is a bustling urban center where modern influences blend with traditional customs. The people of Santiago are renowned for their warm hospitality and strong community ties, evident in their daily interactions and local festivities.

Economically, Santiago Island mirrors Cabo Verde’s broader economic landscape. Agriculture, fishing, and tourism form the backbone of the local economy, supplemented significantly by remittances from the diaspora. Despite economic challenges and regional disparities—with inland regions lagging behind coastal areas in development—the island is making gradual progress. With a population of over 300,000, Santiago is striving towards sustainable development, focusing on improving infrastructure, education, and healthcare. The government’s efforts are directed at bridging the gap between urban and rural areas, fostering economic growth, and enhancing the overall quality of life for its residents.

The study “Cardiovascular Prevention in Cabo Verde—Santiago Island” aimed to estimate the prevalence of risk factors associated with cerebrocardiovascular diseases in the adult population residing on Santiago Island. This is important because of these issues:

High Prevalence of Chronic Diseases: Cerebrocardiovascular diseases are one of the leading causes of morbidity and mortality in Cabo Verde. Understanding the risk factors associated with these conditions is crucial for implementing effective preventive measures.

Changing Epidemiological Profile: Cabo Verde is experiencing an epidemiological transition where non-communicable diseases, such as cerebrocardiovascular diseases, are replacing infectious diseases as the primary causes of illness and death. This study will help quantify and characterize these emerging risk factors.

Public Health Planning and Policy: Understanding the prevalence and distribution of risk factors allows policymakers to develop targeted interventions, allocating resources more effectively for prevention and treatment programs.

Improvement of Quality of Life: Identifying the main risk factors in the adult population of Santiago can lead to public health initiatives that promote lifestyle changes, such as increased physical activity, healthy diets, and reduced tobacco and alcohol consumption, thereby improving the inhabitants’ quality of life.

Data for Future Research: The results of this study will provide a valuable database for future research, helping to monitor trends over time and assess the effectiveness of public health interventions.

## 2. Materials and Methods

### 2.1. Sample

The sample size for this population-based, cross-sectional study was determined using demographic projections for Santiago Island, Cabo Verde, which estimated a population of 317,238 individuals in 2021. To ensure the sample was representative and statistically significant, the following parameters were used in the calculation:

1. Estimated prevalence: The sample size calculation assumed an estimated prevalence of 50%. This prevalence was chosen because it provides the maximum variability in the population, leading to the most conservative and thus larger sample size, ensuring robustness in the study results.
2. Confidence interval: A 95% confidence interval was selected. This level of confidence is standard in research and implies that we are 95% confident that the sample results reflect the true population parameters.
3. Standard error: A standard error of 4% was set to ensure the precision of the estimates. This margin of error is a typical choice for public health studies, balancing the need for accuracy with practical considerations.

Using these parameters, the sample size was calculated to be 599 individuals. This sample was proportionally distributed across the nine municipalities of Santiago Island to ensure representation from each area. Data collection was performed randomly and door-to-door to further enhance the representativeness and minimize sampling bias.

This approach provides a statistically sound basis for the sample size, ensuring that the study’s findings are reliable and generalizable to the population of Santiago Island.

### 2.2. Study Design

Participants were selected according to inclusion criteria: aged over 18, of both sexes, residents of Santiago Island, and willing to participate by signing an informed consent form. Excluded were Cabo Verdean nationals who had stayed abroad for over a year and had arrived on Santiago Island less than 30 days before the study began. Also excluded were individuals with any disability that might have prevented them from participating in the study. For data collection, participants were invited to participate through the reading of the informed consent form, and all procedures were explained. Households were selected by random sampling, and contact with individuals always started from an index house, with data collection proceeding from nearby residences, alternating three or more houses in any direction whenever possible. If more than one individual in a household met the inclusion criteria, data collection procedures were applied to all. Data collection and measurement of various variables were carried out using a CCV risk factors assessment questionnaire. Collected data were recorded in a database for later analysis, including information on age, sex, race, weight, height, presence of diabetes, dyslipidemia, heredity, physical activity, alcoholism, smoking habits, history of CCV events, and weight and blood pressure monitoring habits. Physical exercise was quantified by frequency (days per week) and duration (minutes/hours). Smoking habits were assessed by the number of cigarettes per day and years of use. Alcohol consumption was quantified by the number of drinks per day, either during or outside meals. Regarding a history of CCV pathologies, participants were asked if they had experienced AMI, stroke, and/or TIA. Heredity questions included whether any family member had known heart disease. Participants were also asked about their habits of blood pressure and weight monitoring and their frequency. Weight was measured using a SECA^®^ digital scale, with participants instructed to stand on the scale barefoot, with empty pockets, a straight posture, and arms along the body. Height was measured using a SECA^®^ portable stadiometer placed on a flat surface. Participants were asked to remove any head objects that could interfere with measurement, stand with their backs to the stadiometer, legs and feet parallel, and arms along the body. After aligning the back of the head and straightening the back, the movable part of the stadiometer was moved to the highest point of the head. BMI was calculated using the height measured with the stadiometer and weight measured with the calibrated digital scale. Values were categorized according to WHO guidelines: underweight: <18.5 kg/m^2^; normal weight: 18.5–24.99 kg/m^2^; overweight: 25–29.99 kg/m^2^; obesity grade I: 30–34.99 kg/m^2^; obesity grade II: 35–39.99 kg/m^2^; obesity grade III ≥ 40 kg/m^2^. BMI was obtained using the formula BMI = weight/(height)^2^ [10]. Blood pressure assessment was conducted following the protocol of the Beira Baixa Blood Pressure Program (PPABB) using an OMRON^®^ M3 Healthcare Co., Ltd., Kyoto, Japan automatic device. After completing the questionnaire and recording weight and height, the participant remained seated for a few minutes before the first measurement. Participants were advised to remain comfortably seated, with their arm supported on a flat surface, sitting straight, without talking or moving. The cuff was placed at heart level on the brachial artery, 2–3 cm above the cubital fossa. Three measurements were taken, with additional readings if the first two varied by more than 10 mmHg. Blood pressure values were classified according to the 2023 European Society of Cardiology Guidelines [10,11].

The presence of diabetes was determined by assessing capillary blood glucose levels at the time of questionnaire administration, which also included questions related to this pathology. The assessment was conducted using a FreeStyle^®^ manual device with glucose test strips Abbott Diabetes Care Inc., Alameda, CA, USA. Before collecting the blood sample, the finger to be pricked was disinfected with alcohol-soaked cotton, and single-use disposable needles were used. The values were analyzed according to the data from the Portuguese Diabetes Association [12], considering an individual diabetic if fasting blood glucose was >126 mg/dL or >200 mg/dL two hours after a meal. All equipment was duly validated by suppliers.

Cholesterol was assessed through a questionnaire that included questions about knowledge of cholesterol and triglyceride levels and the history of using medication to reduce these levels. All sample collection was carried out by the same researcher, with the aim of preventing possible different forms of collection, which could lead to the insertion of uncontrollable factors in the collection.

### 2.3. Statistical Analysis

The data collected from the paper questionnaire were transferred to Google Forms, creating a database in Excel, which was then exported to the statistical program IBM SPSS Statistics^®^ (Statistical Package for the Social Sciences) version 20. To understand the normality distribution of the sample, the Kolmogorov–Smirnov normality test was applied to determine whether the variables followed a normal distribution. The results of the analysis were organized in tables and graphs to facilitate their interpretation and subsequent discussion. Associations between variables were analyzed using the chi-square test, with a significance level of *p* ≤ 0.05 and a 95% confidence interval.

## 3. Results

The study sample consisted of 599 individuals of both sexes, all of whom were Black. Females predominated, representing 54.8%, while males constituted 45.2%. The sample was collected from the nine municipalities of Santiago Island. The minimum age of respondents was 18 years, and the maximum age was 90 years, with an average age of 44 years ± 17.06 years. The age group with the highest number of individuals was 18 to 27 years, representing 21% of the total.

In the study of the sociodemographic profile, the level of education was also assessed. It was found that the majority of individuals had a basic education level (54.9%), secondary education 23%, higher education 6.7%, vocational education 4.2%, and no formal education 11.7%, of whom 83% were female.

A BMI value of ≥25 kg/m^2^ was considered a risk factor for CCVD. It was found that 9.3% of respondents were underweight, 48.1% had a normal weight, 27.2% were overweight, and 15.4% were obese.

In relation to BMI and age group, it was observed that underweight and normal weight were more prevalent in the 18 to 27 age group, overweight was more prevalent in the 48 to 57 age group, and obesity had a higher percentage in the 38 to 47 age group. Females had a higher prevalence of overweight (58.3%) and obesity (80.4%), as shown in Table 1. The intersection of BMI with both sex and age revealed a statistically significant relationship with *p* < 0.001.

**Figure 1:**
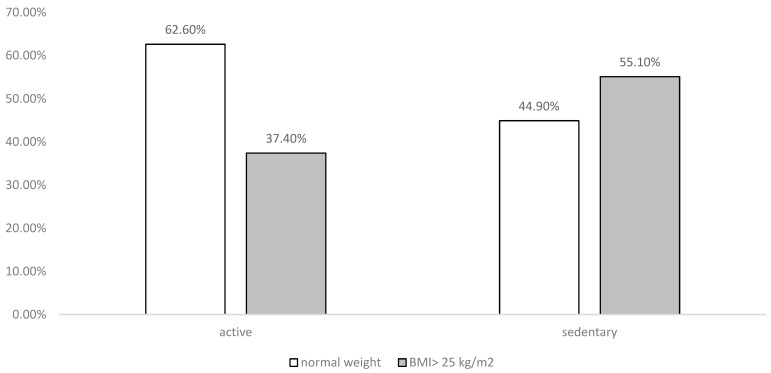
Relationship between physical activity and body mass index.

In relation to the practice of physical activity, it was observed that 34.9% of the sample engages in physical exercise with an average frequency of 4 times per week and an average duration of 59 min and 46 s. Sedentary behavior was more commonly found in females (60.5%), showing a highly significant statistical relationship (*p* < 0.001) (Table 2). Although there was no statistically significant relationship with BMI (*p* = 0.145), it can be perceived from the analysis of Figure 1 that there are more sedentary individuals with a BMI ≥ 25 kg/m^2^ compared to those with normal weight. It was also noticed that the majority of active individuals have normal weight (54.1%).

Considering the risk factor of smoking, it was found that 7.3% were smokers, 3.7% were former smokers, and 4.7% consumed narcotics, with the age group of 28 to 47 years (53.48%) being the group with the highest tobacco consumption. Among the total sample of smokers, the average duration of tobacco consumption was 16.8 years.

When studying alcohol consumption, it was noticed that 14.4% reported having a daily habit of this consumption, and 37.1% had a less frequent habit (only on weekends/festivities). In a more detailed study on this habit, it was found that the daily consumption of alcoholic beverages was approximately 3.7 glasses, of which 92.7% had the habit of doing so both within and outside meals.

When related to the level of education, it was observed that both smoking and alcoholism were more prevalent in individuals with a basic level of education, in the age group of 28 to 47 years, and in males, showing a statistically significant relationship, *p* < 0.001. In Table 2 we can observe three daily habits (sedentary lifestyle, alcoholism and smoking), observing the existence of statistically significant differences by sex (Table 2).

Analyzing the lipid and diabetic profile evaluated through the questionnaire, it was found that 7.5% of individuals reported a diagnosis of diabetes, 8.3% reported a diagnosis of hypercholesterolemia, 1.3% reported hypertriglyceridemia, and 12% reported having had dyslipidemia. Of the 7.5% of diabetic individuals, 11.9% were insulin-dependent, 26.2% followed a diet, and 61.9% were treated with pills. Of the 72 individuals who had hypercholesterolemia, 61.4% adopted pharmacological treatment, 11.4% controlled it only with diet, and 27.1% did not undergo any control. It is observed that diabetes, hypercholesterolemia, hypertriglyceridemia, and a history of hypercholesterolemia presented a higher prevalence in females and in the age group of 58 to 67 years, with a statistically significant relationship, except for hypertriglyceridemia (Table 3).

For the assessment of capillary glycemia, values were classified into hypoglycemia, normal glycemia, pre-diabetes, and diabetes. Glycemia values ranged from 32 mg/dL to 389 mg/dL, with an average of 104.95 mg/dL. Of the assessments performed, 89.8% were post-meal and 10.2% were fasting. It was found that 7.7% had values indicative of pre-diabetes and 4.5% had values corresponding to diabetes. There was a greater predominance of females and the age group of 58 to 67 years, both in pre-diabetes and diabetes.

When cross-referencing diabetes with other risk factors, several statistically significant relationships were observed:

1. Age Group: There is a statistically significant relationship between diabetes and the age group of 58 to 67 years. This suggests that individuals within this age range are more likely to have diabetes compared to other age groups, which can be attributed to various factors such as aging, lifestyle, and genetic predisposition. The statistical significance indicates that this relationship is not due to random chance but rather represents a true association in the population.
2. Hypercholesterolemia and Hypertriglyceridemia: A statistically significant relationship was also found between diabetes and both hypercholesterolemia and hypertriglyceridemia. This finding is consistent with the known medical correlation where dyslipidemia often coexists with diabetes, contributing to cardiovascular risks. The statistical significance reinforces the validity of this association.

However, when examining the educational level of diabetic individuals, it was found that 60.9% have basic education. Despite this observation, there was no statistically significant relationship between diabetes and educational level. This means that the observed distribution of educational levels among diabetic individuals does not differ significantly from what might be expected by chance alone.

To further understand the statistical implications of these risk factors, they were analyzed by sex. This stratification by sex is crucial for identifying any gender-specific patterns or differences in risk factor prevalence and their association with diabetes. By examining these relationships, more targeted and effective public health interventions can be developed to address the needs of different demographic groups. By using the chi-square test, the study ensures that the associations identified are statistically valid and not due to random variation. The results guide public health efforts in targeting specific demographic groups more effectively (Table 4).

The regular habit of assessing blood pressure was recorded in 23.5% of respondents, being more common in females (69.5%) than in males, with 38% reporting that blood pressure values are usually elevated. To determine systolic blood pressure (SBP) and diastolic blood pressure (DBP) values, the average of three blood pressure assessments was calculated. SBP ranged from 211.47 mmHg to 76 mmHg, with an average of 128.47 mmHg, and DBP ranged from 150 mmHg to 51.67 mmHg, with an average of 82.75 mmHg.

It was found that the prevalence of arterial hypertension (AHT) in the adult population of Santiago Island is 32.6%, with 53.8% being females and 46.2% males. In the distribution of hypertensive individuals by AHT classes, it is noted that 34.6% had grade I AHT, 26.2% had grade II AHT, 14.1% had grade III AHT, and 25.1% had isolated systolic AHT.

**Figure 2:**
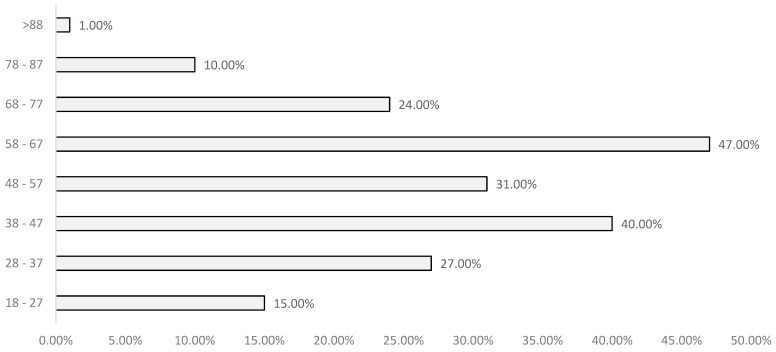
Distribution of arterial hypertension by age group.

According to the distribution of the sample across different age groups, arterial hypertension was found to have the highest prevalence in the group aged 58 to 67 years, followed by those aged 38 to 47 years. This relationship was statistically significant, with a *p*-value of less than 0.001. This pattern is clearly illustrated in Figure 2.

In examining the relationship between arterial hypertension (AHT) and other risk factors, it was concluded that AHT has a statistically significant association with body mass index (BMI), diabetes, age group, and obesity (Table 5). 

Regarding the study of individuals with cerebrocardiovascular diseases (CCVD), it was observed that 3.5% of respondents reported having experienced a transient ischemic attack or stroke, with 66.7% of these cases being females aged between 58 and 67 years. In the study of acute myocardial infarction (MI), three cases (0.5%) were identified, all of whom were female. Further analysis of the antecedents of cerebrocardiovascular pathology and associated risk factors revealed that these individuals had pre-existing conditions such as hypertension, hypercholesterolemia, and diabetes prior to the occurrence of the cardiovascular event. A statistically significant relationship was found between these conditions and risk factors such as hypercholesterolemia, hypertriglyceridemia, and AHT, as detailed in Table 6.

**Figure 3:**
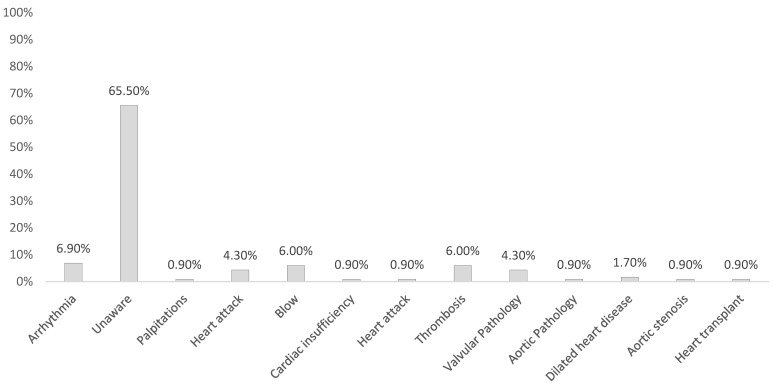
List of cerebrocardiovascular pathologies reported by respondents about their relatives.

The hereditary risk factor was also investigated, revealing that 19.9% of individuals reported having close relatives with cardiovascular and cerebrovascular diseases (CCVD). However, a significant 66% of these individuals could not specify the exact diseases affecting their relatives. In contrast, 34% of respondents were able to identify and report the specific diseases, as illustrated in Figure 3.

The distribution of risk factors by municipalities, as outlined in Table 7, reveals distinct patterns of prevalence for each risk factor within the different municipalities. Obesity is most prevalent in the municipality of São Miguel, while smoking is more common in Tarrafal. Ribeira Grande de Santiago shows higher percentages of sedentary lifestyle, hypertension (AHT), and diabetes. The municipality of São Domingos has the highest prevalence of hypercholesterolemia, and São Salvador do Mundo has the highest rates of hypertriglyceridemia. Furthermore, it was observed that there is a significant relationship between alcoholism, smoking, hypercholesterolemia, and hypertriglyceridemia across all municipalities, indicating widespread public health challenges related to these risk factors.

**Figure 4:**
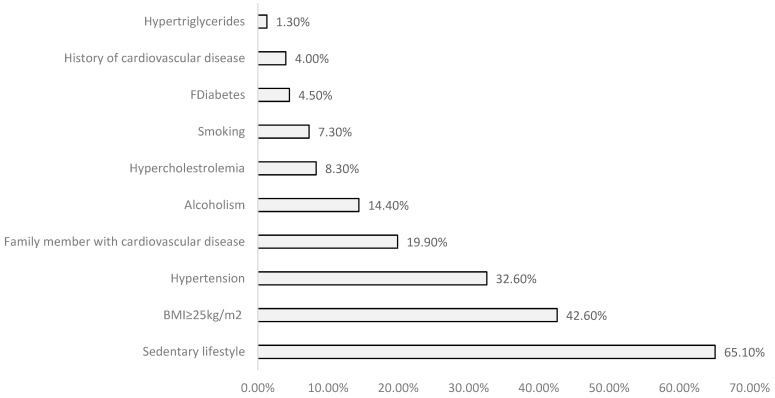
Prevalence of risk factors studied.

From the study of the risk factors analyzed, it is noted that sedentary lifestyle, BMI ≥ 25 kg/m^2^, and hypertension were the ones with the highest prevalence rate (Figure 4).

As a follow-up to this study, a distribution of the number of risk factors for each individual was conducted. It is observed that only a minority have no risk factors for CCVD, and the majority have two or three risk factors, as can be seen in Table 8.

**Figure 5:**
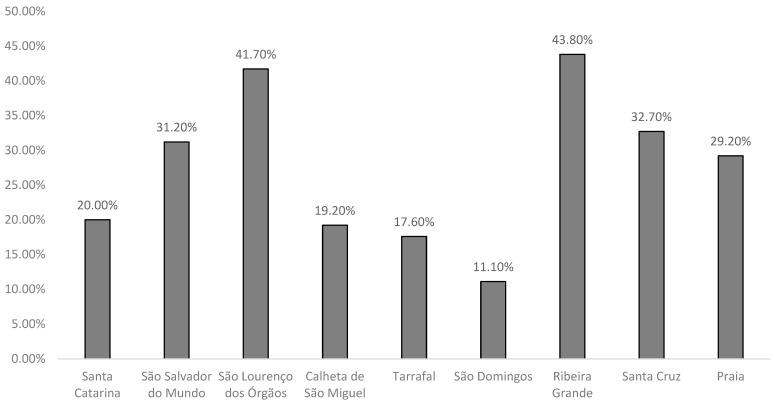
Distribution of individuals with three or more risk factors by municipalities.

When correlating individuals who have three or more risk factors with the various municipalities, it is observed that Ribeira Grande de Santiago has the highest percentage of such individuals, reaching 43.8%. This significant figure indicates that nearly half of the population in this municipality is facing multiple health risks, underscoring the need for targeted public health interventions and resources to address these issues effectively. (Figure 5).

## 4. Discussion

### 4.1. Introduction to Epidemiological Changes

Societies worldwide have been experiencing significant changes in the epidemiological profile, with chronic noncommunicable diseases (NCDs) becoming increasingly prominent in comorbidities and mortality. This shift is a major concern due to the significant socioeconomic and public health impacts. Factors such as physical inactivity, obesity, diabetes, hypertension, alcoholism, and smoking contribute to this change. Cabo Verde has undergone substantial economic and social transformations, resulting in great disparities among the population, which can lead to varied lifestyles influenced by factors like literacy, nutrition, and physical exercise [13,14,15].

### 4.2. Epidemiological Profile and Risk Factors

Each risk factor has a different weight in the development of cardiovascular diseases (CVDs). Understanding and preventing these factors is crucial, as one often influences the development of another, increasing the likelihood of individuals developing these pathologies. According to Statistics on Living Conditions in Cabo Verde for 2015 and 2016, circulatory system diseases are the leading cause of mortality, with a rate of 161.2 per hundred thousand inhabitants, particularly higher among females. Santiago Island, being the largest and most populous area, presents a diverse population with significant urban and rural disparities, offering a comprehensive understanding of the distribution of NCDs and their associated risk factors [16,17,18,19].

### 4.3. Sociodemographic Variables and Risk Factors

Analyzing risk factors in relation to sociodemographic variables such as age, sex, and education level is essential for developing targeted public health interventions to reduce the overall burden of CVDs. The sample for this study, collected from the nine municipalities of Santiago Island, consists of 599 individuals, predominantly female (54.8%), with a predominance in the 18 to 27 age group. Of the total sample, 54.4% had a basic level of education (eight years), and 11.7% were illiterate [20,21].

### 4.4. Education Level and Health Literacy

Studies have shown a relationship between education level and people’s literacy, demonstrating that higher education reduces the risk of developing diseases and modifiable risk factors for CVD. Basic education provides individuals with better capacity to protect themselves from determinants and risk factors, especially those of a behavioral nature. Individuals with low or no literacy have greater difficulty understanding health information and following medical guidelines, which is compounded by precarious jobs and limited access to healthier products and healthcare [22,23,24].

### 4.5. Nutrition and CVDs

Nutrition plays a fundamental role in maintaining health and well-being and is directly related to the development of many pathologies, particularly CVDs. In Cabo Verde, a segment of the population has poor dietary habits influenced by sociocultural, economic, demographic, and lifestyle determinants, leading to overweight and obesity. Obesity is a significant risk factor for CVDs, predisposing individuals to hypertension, diabetes, dyslipidemia, and coronary artery disease [25,26,27].

### 4.6. Body Mass Index (BMI)

The study found that 27.2% of individuals are overweight, and 15.4% are obese, totaling 42.6% with a BMI ≥ 25 kg/m^2^. This is consistent with a national study, IDNT II, where 44.2% of adults were overweight, and 14.3% were obese. A higher prevalence of overweight and obesity was observed in females and older age groups. Comparisons with studies conducted in other regions, such as Sub-Saharan Africa and Portugal, highlight differences in prevalence due to sampling methods, lifestyle factors, and other variables [25,26,28].

### 4.7. Physical Activity and Sedentary Lifestyle

Physical activity is crucial for preventing and controlling risk factors for CVDs. The study categorized individuals as active or sedentary, finding that 34.1% engaged in regular physical exercise, predominantly males. Among sedentary individuals (65.1%), a higher prevalence was observed in females, consistent with findings from the IDNT II report. Comparisons with studies conducted in Angola and Brazil highlight variations in physical activity levels and sedentary behavior due to different daily habits and environmental conditions [29,30,31,32,33].

### 4.8. Alcohol and Tobacco Consumption

Alcohol and tobacco consumption are significant risk factors for CVDs. On Santiago Island, 14.4% of individuals reported alcohol consumption, predominantly males in the 28 to 47 age group. Smoking was observed in 7.3% of adults, mostly males in the same age group. These findings are compared with national and regional data, showing variations in prevalence due to sampling methods and sociodemographic factors [33,34,35,36,37,38].

### 4.9. Lipid Profile

Most individuals reported never undergoing an assessment of their cholesterol and triglyceride levels. The study found 8.3% with hypercholesterolemia and 1.3% with hypertriglyceridemia, predominantly in females and older age groups. Comparisons with other studies highlight differences in prevalence due to data collection methods and the need for blood assessments to evaluate lipid profiles accurately [39,40,41].

### 4.10. Hypertension

Hypertension is influenced by factors such as sedentary lifestyle, obesity, diet, alcoholism, smoking, diabetes, and stress. The study found that 32.6% of respondents had hypertension, predominantly females and older age groups. Comparisons with studies conducted in Guinea-Bissau, Angola, and Brazil show similar high prevalence rates, highlighting the global nature of hypertension as a serious health problem [34,39,41].

### 4.11. Diabetes

Diabetes has become one of the top 10 causes of global mortality, with significant increases since 2000. The study found a diabetes prevalence of 7.5% based on self-reported information and 4.5% based on capillary blood glucose assessment, predominantly in females. Comparisons with national data and studies conducted in Guinea-Bissau and Angola show variations in prevalence due to sampling methods and sociodemographic factors [39,42,43,44].

### 4.12. Cardiovascular Disease (CVD) History

The study found a prevalence of TIA/stroke at 3.5% and AMI at 0.5%, predominantly in females and older age groups. Comparisons with studies conducted on the island of Maio and in Angola show variations in prevalence due to geographical areas, sample sizes, and data collection methods [42,45,46].

### 4.13. Municipality of Ribeira Grande de Santiago

The study found that Ribeira Grande de Santiago had higher proportions of risk factors, sedentary lifestyle, hypertension, and diabetes. No previous studies or data were available for comparison, but it is believed that local dietary habits and alcohol consumption contribute to these findings. Local health teams should focus on specific health literacy interventions for this population.

### 4.14. Sex Differences in Risk Factors

Most risk factors were more prevalent in females, except for smoking and alcoholism. This finding is consistent with studies conducted in Brazil and Portugal [27,33,45]. Women in Cabo Verde play central roles in families and communities but face significant inequalities, impacting their health and lifestyle.

Women in Cabo Verde exhibit a higher prevalence of risk factors for cardiovascular diseases (CVD) due to several interconnected factors.

Social and Cultural Roles:

○ Women often juggle multiple responsibilities, both domestic and professional, which can increase stress levels—a significant risk factor for CVD.
○ As primary caregivers, women may have less time for physical activity and personal healthcare.

Socioeconomic Conditions:

○ Disparities in access to healthcare, education, and well-paying jobs may limit women’s ability to adopt healthy lifestyles.
○ Lower educational levels among women can result in poorer health literacy and less effective management of CVD risk factors.

Lifestyle Factors:

○ Higher rates of physical inactivity among women contribute to obesity, hypertension, and other risk factors for CVD.
○ Dietary habits influenced by cultural and economic constraints may lead to unhealthy eating, exacerbating the risk of CVD.

Health Behaviors:

○ Limited access to health information and preventive services may affect women’s ability to manage risk factors effectively.
○ Women may delay seeking medical care or treatment due to prioritizing family needs over their own health.

Biological and Hormonal Differences:

○ Hormonal changes related to reproductive events, such as menopause, are associated with increased cardiovascular risk.
○ Women often have different risk profiles, with higher prevalence of conditions like obesity and hypertension.

Prevalence of Chronic Diseases:

○ Higher rates of obesity, hypertension, and diabetes among women contribute significantly to their overall CVD risk.

Alcohol and Tobacco Use:

○ While alcohol consumption is more common among men, women who drink may face heightened risks due to social and emotional factors.
○ Although less prevalent, smoking among women also adds to the risk, especially when combined with other factors.

Addressing these issues requires targeted public health policies that focus on education, preventive care, and support for healthier lifestyles, tailored specifically to the needs and challenges faced by women in Cabo Verde.

Support for gender equality policies is crucial to ensure progress and empowerment.

### 4.15. Multiple Risk Factors

The majority of individuals had two or more risk factors for CVD, with a higher prevalence in the 38–47 age group. Comparisons with others studies show similar findings [47,48,49,50,51]. The study highlights the importance of understanding the determinants of multiple risk factors and their relationship with CVDs.

The high prevalence of risk factors and the considerable number of individuals with multiple associated risk factors underscore the need for a deeper understanding of these determinants and their relationship with cerebrocardiovascular diseases. Reinforcing health promotion strategies is essential for individuals, families, and communities to adopt healthy habits and lifestyles and prevent chronic diseases.

Despite all the effort in carrying out the work and its notable added value, it is also necessary to clearly reflect on the potential limitations, with a view to future improvements [45].

### 4.16. Sample Size and Representativeness

Limitation: The study sample of 599 individuals may not fully represent the diverse population of Santiago Island, limiting generalizability.

Impact: Results may not be applicable to the entire population or other regions.

### 4.17. Data Collection Method

Limitation: Data were gathered through self-reports and interviews, which may introduce response bias and inaccuracies.

Impact: Reliance on self-reported data can affect the reliability of results on health behaviors and conditions.

### 4.18. Comparative Data Issues

Limitation: Comparisons with international and regional studies might be influenced by methodological differences.

Impact: Variations in methodology can complicate the interpretation and comparison of findings.

### 4.19. Risk Factor Measurement

Limitation: Many risk factors were assessed based on participant self-reports rather than objective measurements.

Impact: This may affect the accuracy of data on cholesterol levels, diabetes, and other conditions.

### 4.20. Prevalence Variability

Limitation: Prevalence rates of conditions like obesity and diabetes might vary due to differences in study design and diagnostic criteria.

Impact: This could hinder direct comparisons and interpretations of the data.

### 4.21. Sociocultural Differences

Limitation: The study may not have fully accounted for sociocultural differences between urban and rural areas.

Impact: Ignoring these differences can lead to an incomplete understanding of risk factors and health needs.

### 4.22. Sex Analysis

Limitation: While the study highlighted risk factors among women, it may not have fully explored gender-specific differences.

Impact: This may affect the effectiveness of targeted health interventions.

### 4.23. Uncontrolled Confounding Factors

Limitation: Not all potential confounding variables may have been controlled for in the analysis.

Impact: This could affect the validity of causal inferences.

### 4.24. Generalizability of Results

Limitation: Findings may not be generalizable to other regions or populations with different demographics.

Impact: This limits the applicability of the study’s conclusions to broader contexts.

These limitations should be considered when interpreting the results and developing future research and health interventions.

## 5. Conclusions

The results underscore a significant presence of risk factors associated with cerebrovascular diseases among adults on Santiago Island, particularly among females and individuals aged 50 and above. Exceptions include smoking and alcohol consumption, which were more common among males and younger age groups. Among those with a history of cerebrovascular diseases, prevalence remained higher among females and older individuals. Notably, only a small percentage (9.3%) of the population did not exhibit any risk factors.

These findings highlight the urgent need for preventive measures and interventions to mitigate the impact of these risk factors on public health. Disseminating concrete knowledge and raising awareness among competent authorities is essential for implementing necessary improvements.

New investigations and continuous monitoring of potential measures on the island are crucial, given the scale of the problem and its potential to become increasingly uncontrolled. Sharing these results with local health authorities (one of the researchers is part of the local Public Health structure) is key to ensuring that this work contributes positively. This study serves as an initial diagnosis, given the scarcity of similar research in this geographic area, and highlights the need for future policy development. Knowledge dissemination remains the most effective approach.

Concrete applications for the future include developing targeted public health campaigns focused on reducing smoking and alcohol consumption among younger males and implementing routine health screenings for early detection of hypertension and diabetes among older females. Additionally, policies promoting healthier lifestyles through community-based programs and education could significantly reduce the prevalence of these risk factors.

## Figures and Tables

**Table 1:** Distribution of body mass index by age group and sex.

	Age Group (Years)									
	18–27 *n* = 126	28–37 *n* = 98	38–47 *n* = 102	48–57 *n* = 94	58–67 *n* = 62	68–77 *n* = 69	78–87 *n* = 36	>88 *n* = 12	F (%) *n* = 329	M (%) *n* = 270
Underweight (%)	38.3	16.1	17.9	7.1	10.7	5.4	3.6	0	53.6	46.4
Normal Weight (%)	28.8	20.5	18.1	12.5	11.1	5.2	3.5	0.3	53.8	46.2
Overweight (%)	9.2	19.0	19.6	20.9	16.6	11	3.1	0.6	58.3	41.7
Obesity (%)	6.5	25	25	14.1	21.7	4.3	3.3	0	80.4	19.6

Legend: %—percentage, M—male; F—female.

**Table 2:** Distribution of daily habits by sex.

Variables		Total (%)	F (%)	M (%)	*p*
**Sedentary lifestyle**		65.1	60.5	39.5	<0.001
**Smoking**	Smoker	7.3	15.9	84.1	<0.001
	Ex-Smoker	3.7	9.1	90.9	
	Illicit Substances	4.7	50	50	
**Alcoholism**		14.4	4.7	95.3	<0.001

Legend: %—percentage; F—female; M—male.

**Table 3:** Distribution of lipid and diabetic profile by sex and age group.

Variables	Total	F	M	Relation with Sex (*p*)	Relation with AG	Relacion with AG (*p*)
Diabetes	7.5%	71.1%	28.9%	0.013	58–67	<0.001
Hypercholesterolemia	8.3%	80%	20%	<0.001	58–67	<0.001
History of Hypercholesterolemia	12%	77.8%	22.2%	<0.001	58–67	<0.001
Hypertriglyceridemia	1.3%	62.5%	37.5%	0.378	58–67	0.206

Legend: %—percentage; F—female; M—male; AG—Age group.

**Table 4:** Relationship between diabetes and risk factors.

Risk Factors	*p*
Sex	0.862
Obesity	0.146
Sedentary lifestyle	0.664
Age Group (58–67 years)	0.002
Education Level (Basic Education)	0.536
Alcoholism	0.732
Smoking	0.380
Hypercholesterolemia	0.006
Hypertriglyceridemia	<0.001

Legend: *p*—value.

**Table 5:** Relationship between arterial hypertension and risk factors.

Risk Factors	*p*
Sex	0.755
Age Group	<0.001
Body mass index	0.001
Obesity	0.004
Sedentary lifestyle	0.268
Alcoholism	0.234
Smoking	0.307
Diabetes	0.041
Hypercholesterolemia	0.119
Hypertriglyceridemia	0.220

Legend: *p*—value.

**Table 6:** Relationship between history of cardiovascular brain diseases and risk factors.

Risk Factors	Predominance	%	*p*
Sex	Feminine	54.8	0.151
Age group	18–27 years	21	0.174
Body mass index	Normal weight	48.1	0.740
Education level	Basic education	54.4	0.304
Physical activity	Sedentary	65.1	0.740
Smoking	Non-smokers	84.3	0.925
Alcoholism	Non-consumers	48.6	0.094
Hypercholesterolemia	Unknown	56.8	0.007
Hypertriglyceridemia	Unknown	72.1	<0.001
Family with cardiovascular brain diseases	No Illness	58.3	0.701
Arterial hypertension	Without AHT	67.4	0.049
Diabetes	Non-diabetics	96.2	0.366
Individuals with three or more risk factors	Not applicable	73.1	0.144

Legend: AHT—arterial hypertension; %—Percentage; *p*—value.

**Table 7:** Distribution of risk factors by municipalities.

Places										
Variable	P	Sta.C	S.C	T.	S.D	C.S.M	S.S.M	R.G.S	S.L.O	*p*
Obesity	17.6	10	18.4	8.8	14.8	23.1	6.2	6.2	8.3	0.391
Sedentary lifestyle	65.3	58.9	77.6	67.6	40.7	76.9	50	87.5	66.7	0.16
Alcoholism	7.9	11.1	30.6	50	14.8	11.5	18.8	31.2	25	<0.001
Smoking	8.8	7.8	2	29	0	0	0	25	16.7	0.004
Arterial hypertension	34	27.8	24.5	29.4	25.9	34.6	43.8	50	41.7	0.532
Diabetes	4.3	5.6	0	2.9	3.7	3.8	0	6.2	0	0.832
Hypercholesterolemia	9.4	10	4.1	2.9	11.1	3.8	6.2	6.2	8.3	<0.001
Hypertriglyceridemia	0.6	3.3	0	0	3.7	3.8	6.2	0	0	<0.001

Legend: P—Praia; Sta.C—Santa Catarina; S.C—Santa Cruz; T—Tarrafal; S.D—São Domingos; C.S.M—Calheta São Miguel; S.S.M—São Salvador do Mundo; R.G.S—Ribeira Grande de Santiago; S.L.O—São Lourenço dos Órgãos; *p*—value.

**Table 8:** Distribution of numbers of risk factors by individuals.

Distribution of RF	*n*	Total (%)	M (%)	F (%)	Predominance RF
No RF	56	9.3	58.9	41.1	18–27
One RF	165	27.5	47.3	52.7	18–27
Two RF	217	36.2	45.2	54.8	38–47
Three or more RF	161	26.9	38.6	61.4	58–67

Legend: RF—risk factors; *n*—number of individuals; %—percentage; M—male; F—female.

## Data Availability

The original contributions presented in the study are included in the article, further inquiries can be directed to the corresponding author.
